# Hafnium Oxide Nanostructured Thin Films: Electrophoretic Deposition Process and DUV Photolithography Patterning

**DOI:** 10.3390/nano12142334

**Published:** 2022-07-07

**Authors:** Vanessa Proust, Quentin Kirscher, Thi Kim Ngan Nguyen, Lisa Obringer, Kento Ishii, Ludivine Rault, Valérie Demange, David Berthebaud, Naoki Ohashi, Tetsuo Uchikoshi, Dominique Berling, Olivier Soppera, Fabien Grasset

**Affiliations:** 1 CEA, DES, ISEC, DMRC, Université de Montpellier, F-30200 Marcoule, France; 2 Research Center for Functional Materials, National Institute for Materials Science (NIMS), Tsukuba 305-0044, Japan; ishii.kento@nims.go.jp (K.I.); ohashi.naoki@nims.go.jp (N.O.); uchikoshi.tetsuo@nims.go.jp (T.U.); 3 CNRS-Saint Gobain-NIMS, IRL 3629, Laboratory for Innovative Key Materials and Structures (LINK), NIMS, Tsukuba 305-0044, Japan; nguyen.thikimngan@nims.go.jp (T.K.N.N.); david.berthebaud@cnrs.fr (D.B.); 4 Université de Haute-Alsace, CNRS, IS2M UMR 7361, F-68100 Mulhouse, France; quentin.kirscher@uha.fr (Q.K.); lisa.obringer@uha.fr (L.O.); dominique.berling@uha.fr (D.B.); 5 Université de Strasbourg, F-67081 Strasbourg, France; 6 International Center for Young Scientists, ICYS-Sengen, Global Networking Division, NIMS, Tsukuba 305-0047, Japan; 7 Univ Rennes, CNRS, ISCR UMR 6226, ScanMAT UAR 2025, F-35000 Rennes, France; ludivine.rault@univ-rennes1.fr (L.R.); valerie.demange@univ-rennes1.fr (V.D.)

**Keywords:** hafnium oxide, nanoparticles, thin films, nanoarchitectonic, hydrothermal, electrophoretic deposition, DUV, photolithography, micropatterning

## Abstract

In the frame of the nanoarchitectonic concept, the objective of this study was to develop simple and easy methods to ensure the preparation of polymorphic HfO_2_ thin film materials (<200 nm) having the best balance of patterning potential, reproducibility and stability to be used in optical, sensing or electronic fields. The nanostructured HfO_2_ thin films with micropatterns or continuous morphologies were synthesized by two different methods, i.e., the micropatterning of sol-gel solutions by deep ultraviolet (DUV) photolithography or the electrophoretic deposition (EPD) of HfO_2_ nanoparticles (HfO_2-NPs_). Amorphous and monoclinic HfO_2_ micropatterned nanostructured thin films (HfO_2-DUV_) were prepared by using a sol-gel solution precursor (HfO_2-SG_) and spin-coating process following by DUV photolithography, whereas continuous and dense monoclinic HfO_2_ nanostructured thin films (HfO_2-EPD_) were prepared by the direct EPD of HfO_2-NPs_. The HfO_2-NPs_ were prepared by a hydrothermal route and studied through the changing aging temperature, pH and reaction time parameters to produce nanocrystalline particles. Subsequently, based on the colloidal stability study, suspensions of the monoclinic HfO_2-NPs_ with morphologies near spherical, spindle- and rice-like shapes were used to prepare HfO_2-EPD_ thin films on conductive indium-tin oxide-coated glass substrates. Morphology, composition and crystallinity of the HfO_2-NPs_ and thin films were investigated by powder and grazing incidence X-ray diffraction, scanning electron microscopy, transmission electron microscopy and UV-visible spectrophotometry. The EPD and DUV photolithography performances were explored and, in this study, it was clearly demonstrated that these two complementary methods are suitable, simple and effective processes to prepare controllable and tunable HfO_2_ nanostructures as with homogeneous, dense or micropatterned structures.

## 1. Introduction

Materials based on hafnium oxide (HfO_2_) offer remarkable combinations of ferroelectricity, high dielectric permittivity, high energy barriers and high thermodynamic stability, which are of particular interest for next-generation high-κ gate dielectrics in microelectronics or nonvolatile memories, variable capacitors, biosensors, actuators and energy storage/harvesting devices [1,2,3,4]. These chemical and physical properties are known to be highly dependent on the presence of atomic defects and the amorphous nature or various crystal structures of HfO_2_, i.e., monoclinic (space group *P*2_1_/*c*, the most stable at low temperature), tetragonal (*P*4_2_/*nmc*), orthorhombic (*Pca*2_1_) and cubic (*Fm*3*m*) [5,6,7,8]. Recently, metal oxide materials such as ZnO, TiO_2_ and HfO_2_ have emerged as alternatives to conventional organic photoresists and have been shown to be well suited to deep UV and extreme UV (DUV and EUV) photolithography processes [9,10,11,12,13,14,15,16]. Photoresists based on these inorganic materials can be produced as very thin films and can help to push resolution limits down to the nanoscale. In particular, the etch resistance of HfO_2_ nanoparticles is high enough for the production of thinner films with low absorbance and the high chemical and thermal stability and attractive optical properties of HfO_2_ make it suitable for photolithography patterning [17,18,19]. The preparation of HfO_2_ thin films has been widely studied over the past few decades using a variety of techniques such as atomic layer deposition, pulsed laser deposition, radio frequency sputtering, plasma oxidation of Hf films, chemical vapor deposition, spin- or dip-coating, spray pyrolysis, sol-gel techniques or electrophoretic deposition (EPD) [4,20].

In this study, we followed a nanoarchitectonics approach [21,22] to further characterize and potentially optimize nanostructured HfO_2_ films. We investigated the microstructural properties of polymorphic HfO_2_ thin films (<200 nm) prepared using two complementary methods: micropatterning of sol-gel solutions by DUV photolithography and direct electrophoretic deposition (EPD) of HfO_2_ nanoparticles (HfO_2-NPs_). Amorphous and monoclinic micropatterned HfO_2_ thin films (HfO_2-DUV_) were prepared by using a sol-gel solution precursor (HfO_2-SG_) and spin-coating followed by DUV photolithography, whereas continuous and dense monoclinic HfO_2_ thin films (HfO_2-EPD_) were prepared by direct EPD of colloidal dispersions of hydrothermally grown HfO_2-NPs_ [23,24,25,26].

## 2. Materials and Methods

### 2.1. Materials

Hafnium tetrachloride (HfCl_4_, 98.0%) was obtained from Alfa Aesar, sodium hydroxide (AR, 96.0%) and isopropanol were purchased from FUJIFILM Wako Pure Chemical Corporation and acetylacetone (≥99.0% (GC)) was obtained from Nacalai tesque Co. Ltd. Commercial indium-tin oxide-coated glass (ITO glass) substrates (100 × 100 × 1.1 t, 10 Ω/sq) were purchased from Geomatec. Hafnium (IV) tetraisopropoxide (99.9% trace metal basis), methacrylic acid (MAA, 99%) and 1-propanol (anhydrous grade, 99.7%) were purchased from Aldrich. All material preparation steps (for the sol-gel solution, colloidal dispersion and thin films) and the lithography were carried out under atmospheric pressure at room temperature. All chemicals were used as received and deionized water was used throughout the study, for the preparation of the aqueous solutions and during hydrothermal treatments.

### 2.2. Synthesis of the Sol-Gel Solutions and the Monoclinic HfO_2_ Nanocrystals

HfO_2-SG_: The sol-gel solutions were prepared using an optimized method derived from Stehlin et al.’s [10,11]. Hafnium(IV) tetraisopropoxide (5 mL) was mixed with methacrylic acid (MAA) at a M:MAA molar ratio of 1:8. After 5 min of stirring, 2 mL 1-propanol was added as a solvent and after 10 min of stirring, deionized water was added for a M:H_2_O molar ratio of 1:10. The mixture was stirred for 24–48 h before use. The solution was diluted 2-fold with 1-propanol to tune its viscosity and thereby control the thickness of the thin films obtained by spin-coating. The prepared solutions remained stable for several months with no noticeable changes including their photosensitivity.

HfO_2-NPs_: The HfO_2-NPs_ samples were synthesized using a hydrothermal process from hafnium tetrachloride (HfCl_4_) as a starting material [26]. Hafnium hydroxide chloride was prepared by dissolving HfCl_4_ in deionized water to produce Hf(OH)_2_Cl_2_ and Hf(OH)_4_ in a solution at pH = 2. A 1 M aqueous solution of NaOH was added dropwise to increase the pH up to 11.5. The solution was then sealed in a 300 mL Teflon-lined autoclave and heated to 120–180 °C for 2–48 h to produce nanoparticles. The autoclave was left to cool to room temperature and the precipitate was separated by centrifugation and washed with distilled water and ethanol several times under sonication. The final product was dried under vacuum at room temperature or dispersed in acetone, ethanol or an equimolar mixture of acidic ethanol and acetylacetone (at pH 2, adjusted by adding HNO_3_). The dispersions in acetone and ethanol were stable and transparent with solid contents as low as 0.1 wt.%. The dispersion in acidic ethanol and acetylacetone remained stable for several months.

### 2.3. Preparation of the Polymorphic Nanostructured HfO_2_ Thin Films

HfO_2-DUV_: Thin films for lithography with a typical thickness of 100 nm were deposited by spin-coating (3000 rpm, 60 s) and the films were irradiated without pretreatment using an ArF 193 nm laser (Excistar, Coherent). Photopatterning was achieved by contact photolithography using chromium binary masks on fused silica substrates. After irradiation, the thin films were developed for 30 s in cyclohexanone to remove the non-irradiated part. Some films were then annealed at 400 °C or 600 °C for 1 h in air.

HfO_2-EPD_: To fabricate dense and continuous HfO_2_ films by EPD, 0.1 wt.% solutions of HfO_2-NPs_ were prepared in acetone, ethanol or ethanol/acetylacetone (as described above) and then deposited on ITO glass surfaces. The deposition time and applied voltage were adjusted to obtain dense, transparent, homogeneous films. Smooth ITO glass supports were used as anodic substrates and the EPD system (2400 Series SourceMeter, Keithley Instruments Inc., Solon, OH, USA) was connected to a stainless-steel cathode. The distance between both electrodes was fixed at 5 mm. The coatings were then dried in air and kept at a room temperature for 24 h to ensure the solvent had fully evaporated.

### 2.4. Characterization and Analytical Techniques

Powder X-ray diffraction (PXRD) patterns were recorded on a Miniflex 600 diffractometer (Rigaku Corp., Tokyo, Japan) operated at 40 mA and 40 kV, using copper K_α_ radiation (λ = 1.5406 Å). The diffraction angle (2θ) varied from 10° to 70° in 0.02° increments. Crystalline phases were identified in comparison with reference powder diffraction files (pdfs) from the International Centre for Diffraction Data^®^ (ICDD^®^). Grazing incidence X-ray diffraction (GIXRD) patterns of the thin films were measured on a SmartLab diffractometer (Rigaku Corp., Tokyo, Japan) with copper Kα radiation (λ = 1.5406 Å), operated at 50 mA and 40 kV. The angle of incidence (ω) was set between 0.2 and 0.8°. The morphology and microstructure of the films were studied by scanning electron microscopy (SEM) using a JSM-6500F (JEOL, Ltd., Tokyo, Japan) device, equipped with an Oxford INCA x-sight energy dispersive spectroscopy (EDS) detector (JSD-2300), and by transmission electron microscopy (TEM) using a JEM 2100 HR (JEOL, Ltd., Tokyo, Japan) instrument equipped with an Oxford X-Max 80T detector. The zeta potential of the HfO_2-NPs_ suspension was measured using a Zetasizer Nano Z analyzer (Malvern Instruments Ltd., Malvern, UK). Optical properties were determined from absorbance and reflectance measurements at 340 nm using V-650 and V-750 Jasco spectrophotometers. The thickness and refractive index at 633 nm of the HfO_2-SG_ thin films were determined by spectroscopic ellipsometry (UVISEL ellipsometer, HORIBA Jobin Yvon, HORIBA). The experimental data were fit from 1.5 to 6.5 eV with a Tauc–Lorentz model. Infrared spectra of the thin films were acquired through Si substrates on a modified FTIR spectrometer in transmission mode (Nicolet 8700 FTIR, Thermo Fisher Scientific). The microstructured films were characterized using atomic force microscopy (AFM) in the tapping mode, using a PicoPlus 5500 System model from Agilent. Scanning was performed at 1 line/sec with an image resolution of 512 × 512 pixels.

## 3. Results and Discussion

Nanoarchitectonics is an approach in which nanotechnology is combined with other scientific disciplines, such as materials science, organic chemistry, supramolecular chemistry and biotechnology, to synthesize functional materials [21,22]. Through a bottom-up approach, nanosized structural units, usually a group of atoms or molecules, are organized to produce functional materials. This study of nanostructured HfO_2_ thin films follows the nanoarchitectonics concept through it’s a combination of several chemical solution processes with thin film nanotechnology and DUV photolithography to produce different kinds of thin films with a controlled microstructure.

### 3.1. Amorphous and Monoclinic HfO_2-DUV_ Micropatterned Thin Films

Photosensitive sol-gel solutions based on metal-oxo clusters which can be crosslinked and mineralized by DUV irradiation (193 nm) have been shown to be a versatile, fast and simple means of preparing patterned films using exclusively optical methods [10,11]. The procedure generally involves the production of metal-oxo clusters by the complexation of a metal alkoxide with carboxylic acids followed by controlled hydrolysis–condensation.

**Figure 1:**
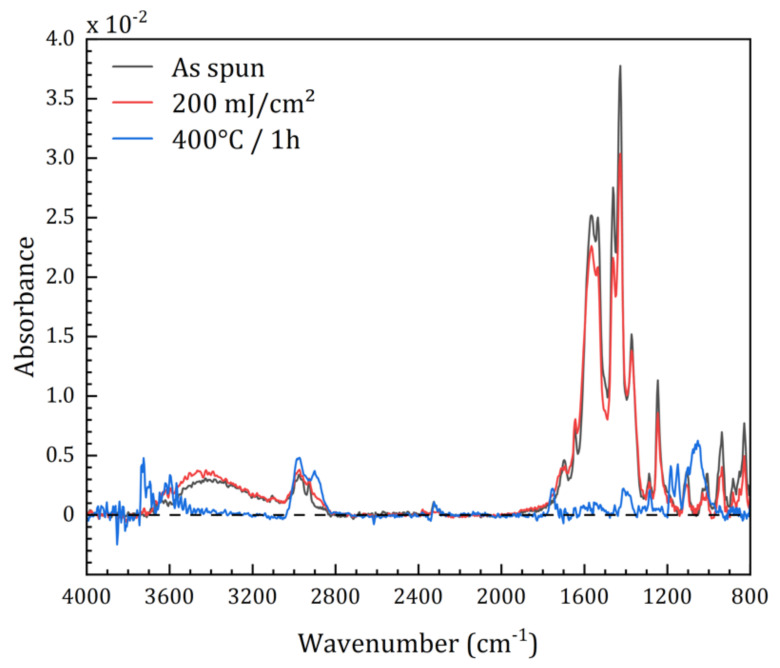
Transmission FTIR spectra of HfO_2-DUV_ thin films deposited by spin-coating and analyzed after deposition, after DUV irradiation (200 mJ/cm^2^) and after heat treatment at 400 °C.

This method has been used with TiO_2_, ZrO_2_ and ZnO, but so far never with HfO_2_. Figure 1 shows how the FTIR spectrum of a HfO_2_-_SG_ thin film changes after processing by DUV photolithography and heat treatment at 400 °C. No significant change was recorded for the higher temperature (600 °C). The FTIR spectrum of the spin-coated film highlights the presence of Hf clusters, with characteristic carboxylate bands at around 1450 cm^−1^ (ν_asym_(COO)) and 1570 cm^−1^ (ν_sym_(COO)) from the methacrylate ligands bonded to Hf centers [27,28,29]. The broad band centered at 3400 cm^−1^ corresponds to hydroxyl groups.

The intensity of the bands in the 1200–1800 cm^−1^ region decreases slightly after irradiation with a moderate dose (corresponding to the dose used to photopattern the film), and the band at 3400 cm^−1^ becomes slightly more intense, indicating an increased concentration of hydroxyls. This suggests that the Hf clusters are partially photolyzed, as observed previously for Ti [11].

The photolysis yield of the clusters is low under these moderate irradiation conditions, as evidenced by the small change in the FTIR spectrum. In contrast, after heat treatment at 400 °C or 600 °C, the FTIR bands disappear completely, which is expected since at these temperatures the sol-gel layer is mineralized.

**Figure 2:**
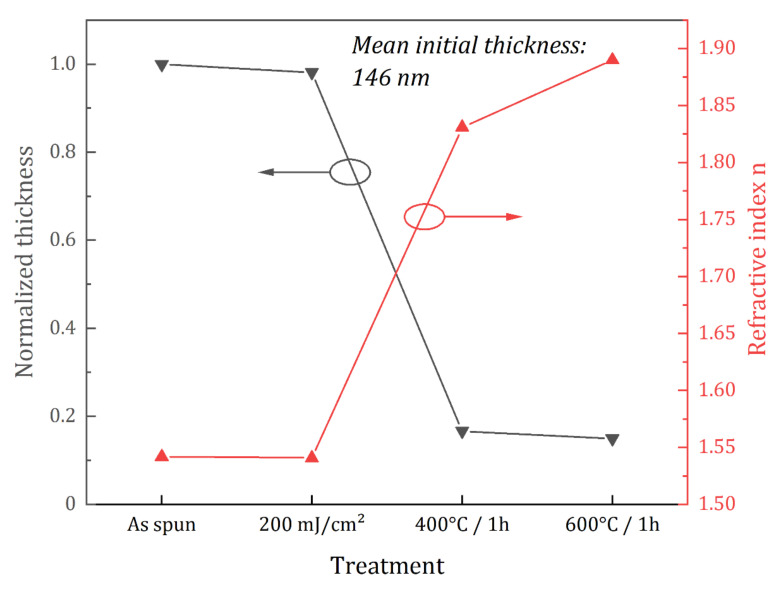
Spectroscopic ellipsometry measurements of the thickness and refractive index of HfO_2-DUV_ thin films after deposition, after DUV exposure (200 mJ/cm^2^) and after DUV exposure and thermal annealing (400 °C or 600 °C).

The values obtained by ellipsometry for the thickness of the films are in agreement with the FTIR data. As shown in Figure 2, the decrease in thickness is only slight during DUV treatment (about 2%), but more substantial during the thermal treatments (85% and 88% at 400 °C and 600 °C, respectively). These sharp decreases correspond to the loss of the organic part of the films contained in the Hf clusters and to condensation reactions leading to the formation of the oxide. As the thin films shrink, they become denser as shown by the increase in the refractive from 1.541 for the as-spun and DUV-treated films up to 1.890 for the films annealed at 600 °C.

**Figure 3:**
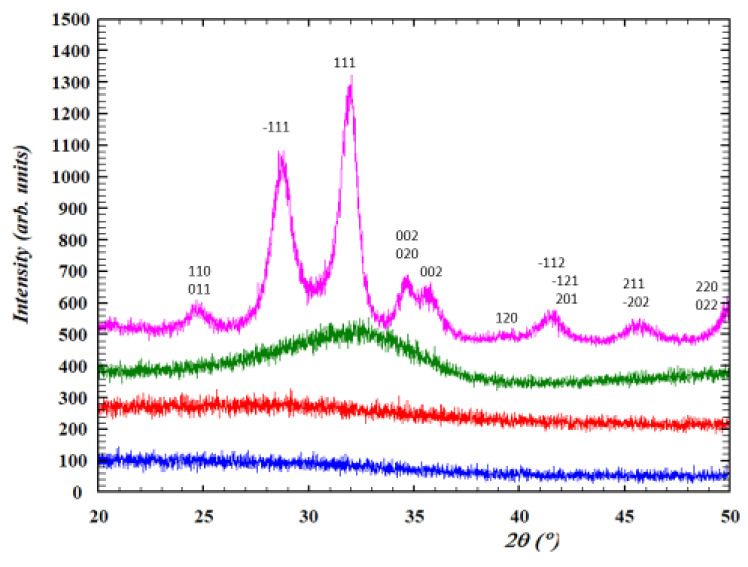
Grazing incidence X-ray diffraction patterns (ω = 0.4°) of HfO_2-DUV_ thin films as-deposited (blue), after DUV treatment at 200 mJ/cm^2^ (red) and after DUV treatment and thermal annealing at 400 °C for 1 h (green) or 600 °C for 1 h (pink).

The featureless GIXRD patterns in Figure 3 show that, as expected, the spin-coated films are amorphous and remain amorphous after DUV treatment and annealing at 400 °C. In contrast, the broad peaks observed after annealing at 600 °C correspond to monoclinic HfO_2_, which is the stable form of crystalline HfO_2_ at this temperature [26,30,31].

**Figure 4:**
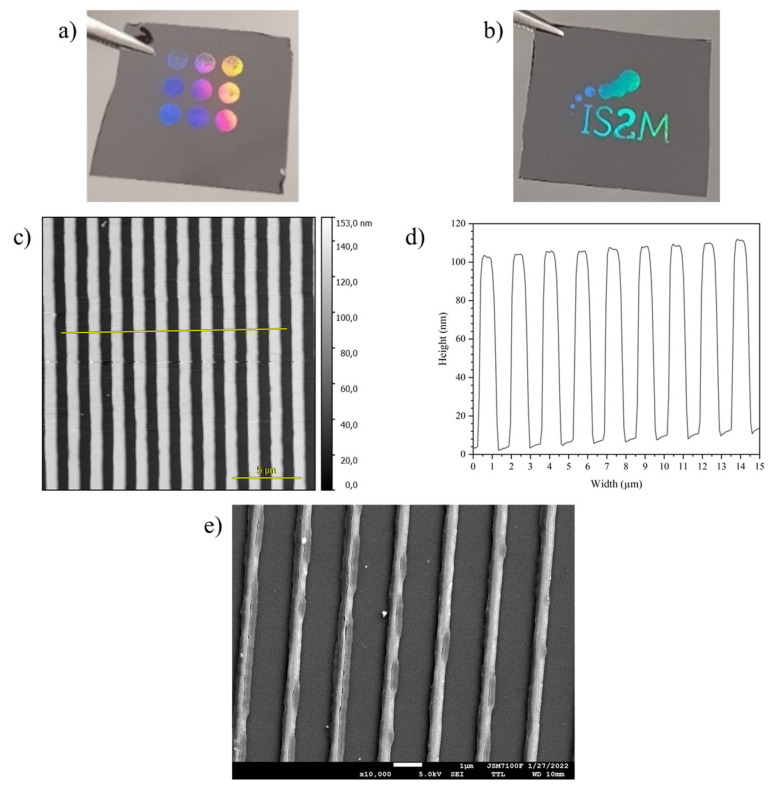
HfO_2-DUV_ thin films patterned by deep UV lithography. (**a**) Photograph of a film with a periodic pattern (1.6 µm period). The circles were printed with doses ranging from 100 to 300 mJ/cm^2^ (in steps of 25 mJ/cm^2^). (**b**) Photograph of a periodic pattern (1.6 µm period) written through a mask to draw the IS2M logo (200 mJ/cm^2^). (**c**) Atomic force microscopy image of the 1.6 µm period patterns (200 mJ/cm^2^) and (**d**) cross-section corresponding to the red segment in part c. (**e**) SEM image of the patterned sample after thermal annealing at 600 °C for 1 h.

The interest of combining these solutions with DUV is that it allows direct patterning, as highlighted in Figure 4.

Figure 4a shows that patterns can be obtained with a wide range of doses around 200 mJ/cm^2^ and Figure 4b shows that macroscopic images can be produced using an additional mask. The atomic force microscopy images in Figure 4c,d show that the patterns are well defined, with low edge roughness, vertical edges and definition down to the Si substrate without any residual layer between the features. This shows that the DUV irradiation induces sufficient crosslinking and densification for the material to resist development. At the molecular scale, crosslinking corresponds to the partial removal of ligands and the production of reactive hydroxyl groups. Irradiated clusters can react with each other by condensation, leading to the formation of the patterns, while non-exposed parts are sufficiently stable to be fully removed by development. These properties indicate that the HfO_2-SG_ solution can be used as a negative tone photoresist.

Figure 4e shows that the patterns are preserved after thermal annealing at a temperature high enough for the HfO_2_ material to crystallize (600 °C for 1 h). This approach is therefore a very simple means to obtain micropatterned HfO_2_ without etching. As expected from the ellipsometry data, however, significant shrinkage occurs at 600 °C, and the patterns become narrower as a result (Figure 4e).

In another approach, we added HfO_2-NPs_ to this HfO_2-SG_ solution in an attempt to pre-crystallize the HfO_2-DUV_ thin films. The modified sol-gel solutions were deposited at room temperature on silicon substrates by spin-coating, but no HfO_2_ phases were detected in the corresponding GIXRD of the coating’s surface (as deposited or annealed at 400 °C or 600 °C for 1 h), either when 5 nm HfO_2-NPs_ or when 40 nm HfO_2-_np_s_ were added to the sol-gel solution (data not shown). This may be because the nanoparticle concentration was too low or because the 5 nm HfO_2-NPs_ are dissolved by the sol-gel solution after addition. Further investigations are in progress to understand this result.

### 3.2. Continuous, Dense, Monoclinic HfO_2-EPD_ Thin Films

Electrophoretic deposition is an attractive colloidal process offering short operation times, high deposition rates, versatility in the thickness and morphology of the deposited layer and scalability in terms of surface size and production volume. EPD has already successfully been used to deposit oxide nanoparticles, sulfide nanoparticles, metal nanoclusters and carbon nanotube thin films on a variety of substrates, sometimes followed by thermal treatment to improve adhesion [32,33,34,35,36,37]. The principal driving force in EPD is the charge of the colloidal particles, whose migration under an applied electric field, leads to the deposition of a homogeneous film. A well-dispersed and stable suspension is therefore essential and several operating parameters such as the voltage, intensity and deposition time need to be adjusted to ensure high-quality thin films are reproducibly obtained [38,39,40]. Here, the solvent type and zeta potential of the solution were also studied as these parameters are known to affect the quality and thickness of the deposited films [40,41]. Nanocrystalline HfO_2-NPs_ with different crystal sizes were therefore synthesized to prepare colloidal suspensions for EPD, and this process was investigated as a potentially simpler route for the preparation of HfO_2_ thin films.

In the first step of this process, nanocrystalline HfO_2-NPs_ were grown using the hydrothermal method [26], and the effects on the size of the particles of temperature, pH and aging time were investigated.

**Figure 5:**
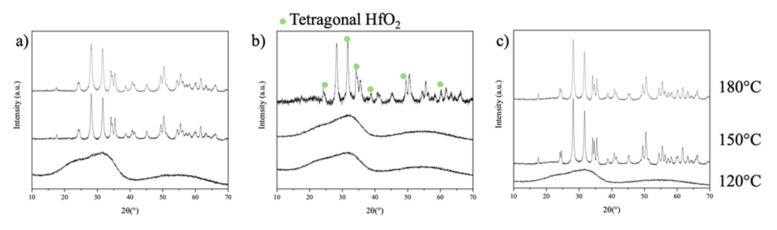
Powder X-ray diffraction patterns of HfO_2-NPs_ prepared by hydrothermal synthesis at 120–180 °C and (**a**) pH 2, (**b**) pH 7 or (**c**) pH 11.5 for 6 h.

Figure 5 shows the PXRD patterns of HfO_2-NPs_ samples prepared at different pHs and temperatures. The absence of diffraction peaks for the nanoparticles prepared at 120 °C indicates that no crystallization occurs at this temperature at any pH. In contrast, crystalline HfO_2-NPs_ form at all pHs at 180 °C and at pH 2 and 11.5 at 150 °C. In each case, the diffraction peaks match those of the monoclinic HfO_2_ [26]. The peaks from the samples prepared at 180 °C are narrower and more intense, indicating higher crystallinity and suggesting that the crystallites are more thermodynamically stable and grow larger at this temperature. The diffraction pattern obtained for the HfO_2-NPs_ prepared at 180 °C and pH 7 (Figure 5b) shows the major characteristic peaks of monoclinic HfO_2_ as well as additional weaker peaks at 31.7°, 34.7°, 50.5° and 60.2°, which suggest that small amounts of tetragonal HfO_2_ form under these conditions [26].

**Figure 6:**
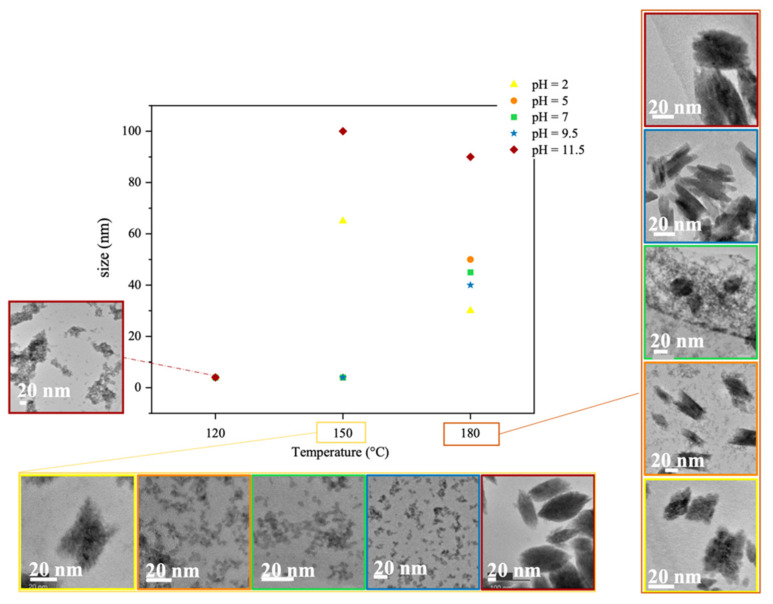
HfO_2_NPs_ size after 6 h of hydrothermal growth at different temperatures and pHs, with corresponding transmission electron bright field micrographs. At 150 °C, three points (orange, blue and green) are superimposed.

**Figure 7:**
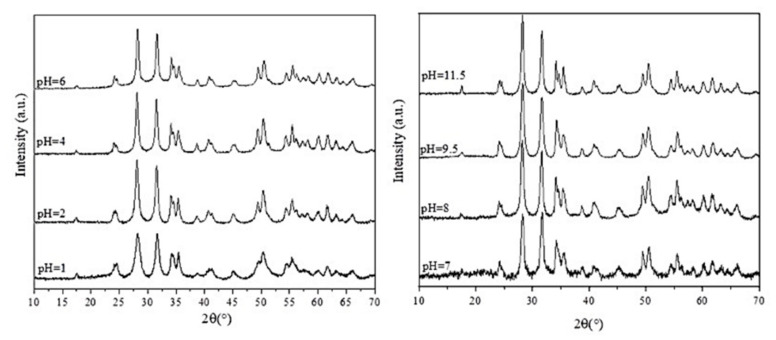
Powder X-ray diffraction patterns of HfO_2-NPs_ prepared by hydrothermal synthesis at 180 °C and pH 1–11.5 for 6 h.

Figure 6 shows how the size of the nanoparticles varies with the synthesis temperature and pH, with corresponding TEM images of their morphology. The amorphous nanoparticles formed at 120 °C are all similarly near-spherical in shape. At pH 2, the nanoparticles are X-shaped, presumably because of the absence of NaOH in the solution, and larger at 150 °C (70 nm) than at 180 °C (30 nm). At 180 °C, secondary particles form at pH 5 and pH 7. The hydroxylation reactions that occur during synthesis depend on the pH. At 25 °C and acidic pHs, the main hydrolyzed hafnium complex is Hf(OH)_3_^+^, while in alkaline solutions (pH 8–11), Hf(OH)_5_^−^ is the major species, and near neutral pH, Hf(OH)_4_ and Hf(OH)_5_^−^ coexist [26]. These hydroxylated complexes are unstable in solution and can promote the simultaneous formation of both tetragonal and monoclinic phases [26]. As the reactions progress at different rates depending on the temperature, the most thermodynamically stable phase at that temperature ends up predominating. At pH 9.5, spindle-like nanoparticles about 50 nm in length are formed, while at pH 11.5, the particles that form resemble grains of rice and are 80–100 nm in size. These results suggest that higher temperatures and pHs lead to the formation of larger, more elongated nanoparticles. The PXRD patterns of HfO_2-NPs_ samples synthesized at 180 °C and pH 1–11.5 (Figure 7) confirm the effects of these two parameters. The diffraction peaks from the monoclinic phase are weaker around neutral pH, indicating the formation of a minor tetragonal phase, whose growth is limited by its thermodynamic stability. Since the reaction time is fixed, the size of the crystallites depends mainly on temperature. The average crystallite sizes estimated from the full width at half maximum of the diffraction peaks using the Scherrer equation are 14 nm (±1 nm), 16 nm (±1 nm) and 18 nm (±1 nm) for the nanoparticles synthesized at 180 °C and pH 2, 9.5 and 11.5, respectively, indicating that the nanoparticles observed by TEM (Figure 6) are polycrystalline and somewhat aggregated.

**Figure 8:**
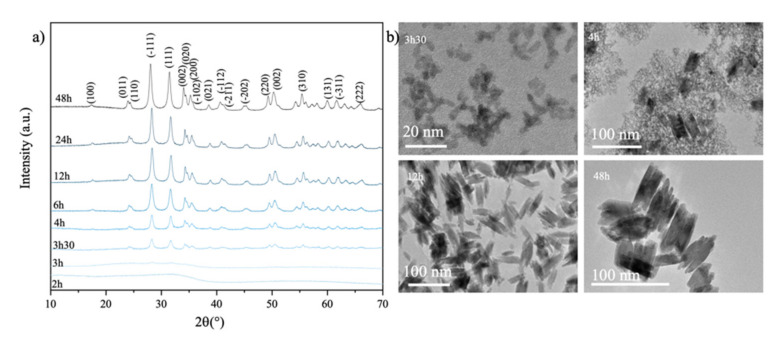
(**a**) Powder X-ray diffraction pattern of HfO_2-NPs_ synthesized at 180 °C and pH 9.5 for 2–48 h. (**b**) Transmission electron bright field micrographs of the nanoparticles after 3.5, 4, 12 and 48 h of hydrothermal growth.

Figure 8a shows how the PXRD patterns of HfO_2-NPs_ synthesized at 180 °C and pH 9.5 evolve as a function of the reaction time. The diffraction peaks become more intense, indicating that the nanoparticles become more crystalline. The peaks all correspond to the monoclinic phase, indicating that this is the only one that forms. The TEM images in Figure 8b show that the particles are near-spherical and about 5 nm in diameter after 3.5 h of reaction time, which is also the minimum size of the crystalline nanoparticles as shown in Figure 8b. These observations suggest that the particles crystallize by heterogeneous nucleation, with large particles surrounded by smaller ones, all with a monoclinic structure. This is consistent with the predominance of Hf(OH)_5_^−^ complexes at this alkaline pH. The particles become more uniform in size at later times, with large spindle-like particles growing by coalescence or Ostwald ripening. After 48 h growth, the particles reached a size of about 60 nm at pH 9.5 and 100 nm at pH 11.5, which is therefore the maximum stable particle size at 180 °C.

**Figure 9:**
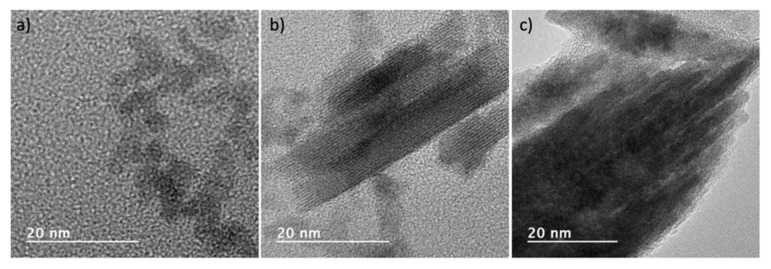
Transmission electron bright field micrographs showing the d-spacing of (**a**) 5, (**b**) 50 and (**c**) 100 nm particles.

Figure 9 shows the lattice images of isolated crystals grown for different times under alkaline conditions (180 °C, pH 9.5 or 11.5) to obtain particles 5, 50 and 100 nm in size. The well-resolved crystal fringes show that the three differently sized nanoparticles are all highly crystalline.

**Figure 10:**
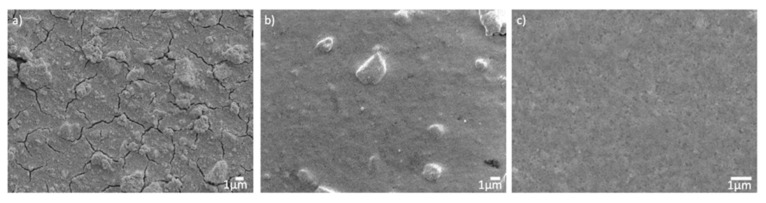
Scanning electron micrographs of the HfO_2-EPD_ films prepared by electrophoretic deposition of colloidal suspensions of HfO_2-NPs_ in (**a**) acetone, (**b**) ethanol or (**c**) an equimolar mixture of ethanol and acetylacetone.

The three types of HfO_2-NPs_ were then directly used to prepare thin films by EPD, without any functionalization of their surface with ligands. SEM images of the coatings obtained by EPD on ITO substrates using dispersions of the nanoparticles in acetone, ethanol and acidic ethanol/acetylacetone are shown in Figure 10. Zeta potential measurements prior to the EPD process showed that the surface charge of the particles was positive, and thus deposition occurred at the cathode. The zeta potential is a commonly used measure of the stability of colloidal suspensions, with low zeta potentials tending to be associated with instability, while beyond 25 or −25 mV, the electrostatic repulsion between the particles stabilizes the suspension against coagulation. The zeta potential is therefore also a useful predictor of the uniformity of the electrophoretic coatings. Indeed, the cracks and agglomerates observed in the coating prepared with the suspension in acetone (Figure 10a) are in keeping with the low zeta potential (~+10 mV) of the colloid. The porosity of the surface and the size of the aggregates also increased with the applied voltage and the deposition rate (data not shown) and increasing the acidity of the dispersion did not improve its stability. The zeta potential of the dispersion in ethanol was much higher (+50 mV), resulting in a more uniform EPD film (Figure 10b) with much fewer cracks and aggregates. The suspensions in the acidic mixture of ethanol and acetylacetone proved to be even more stable over time, with lower sedimentation heights than acetone- or ethanol-based suspensions. This is reflected in a smooth, defect-free coating with tightly packed particles (Figure 10c).

**Figure 11:**
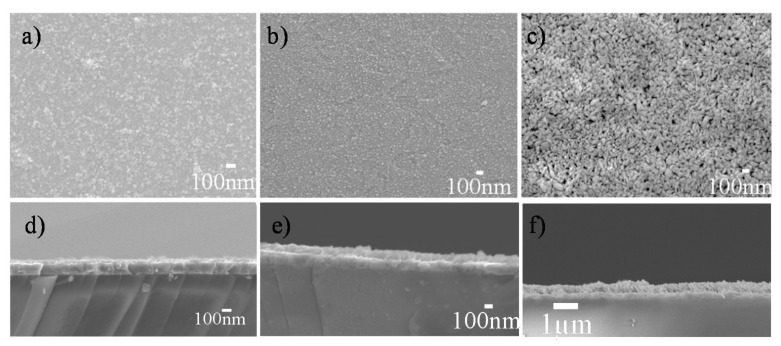
(**a**–**c**) Surface and (**d**–**f**) cross-sectional SEM images of HfO_2-EPD_ coatings prepared by EPD (5 V, 0.7 mA/cm^2^, 90 s) on ITO glass substrates using suspensions of (**a**,**d**) 5 nm, (**b**,**e**) 50 nm, and (**c**,**f**) 100 nm-sized HfO_2-NPs_ dispersed in an equimolar mixture of ethanol and acetylacetone at pH 2.

Figure 11 and Figure S1 compares the macroscopic appearance, microstructure and the thickness of EPD’s coatings prepared with HfO_2-NPs_ of different sizes. All the films are highly transparent (Figure S1) and the deposited particles completely cover the substrate (Figure 11a–c). Deposition occurred at applied voltages above 2 V and high-quality coatings were obtained at low to moderate voltages (5 V for the films shown in Figure 11), which ensured the dispersions remained stable and avoided turbulence. The coatings deposited at voltages above 50 V were of poorer quality, with large agglomerates, holes and a loss of transparency (data not shown). At lower electric fields, the thickness of the coating increased over time and therefore the current density decreased. While the smallest nanoparticles (5 nm) aggregated somewhat during EPD, and the deposited film is not fully uniform (Figure 11a), the film prepared with 50 nm particles is dense and homogeneous (Figure 11b). Meanwhile, the porous nature of the oxide layer obtained with 100 nm particles (Figure 11c) can be explained by their larger size and elongated shape. After 90 s of EPD, the average film thickness (estimated from 10 cross-sectional SEM images) was 45, 100 and 150 nm respectively with the 5, 50 and 100 nm nanoparticles, respectively (Figure 11d–f), the thickness of the film increasing with the size of the HfO_2-NPs_, as expected. The values of the average roughness were estimated by AFM to be around 10 nm (Table S1 and Figure S2). This EPD method can therefore be used to create thin films with specific morphologies and thicknesses by adjusting the composition of colloidal dispersion and the deposition parameters.

**Figure 12:**
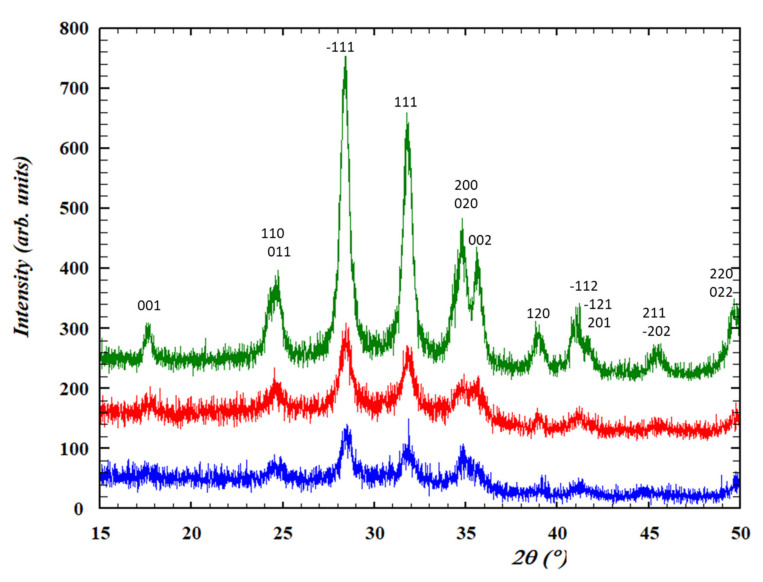
Grazing incidence X-ray diffraction patterns of electrophoretic HfO_2-EPD_ coatings of (blue) 5 nm, (red) 50 nm, and (green) 100 nm sized particles at incidence angles of 0.2° from the surface.

In Figure 12, the GIXRD patterns from the thin films prepared with 5 and 50 nm particles show clear HfO_2_ peaks at an incidence angle of 0.2°. The HfO_2_ signal become weaker at higher angles, and almost negligible at 0.8° because of the thinness of the film (<100 nm) (Figure S3). As expected, HfO_2_ peaks are only observed up to an incidence angle of 0.8° for the film from 100 nm particles with the presence of ITO peaks from the substrate. These results suggest that dense HfO_2_ thin films with different particle sizes can be produced with suitable optoelectronic properties for photolithography patterning.

## 4. Conclusions

In this study, different chemical solution processes were successfully combined with DUV photolithography and EPD to synthesize functional thin film materials. Amorphous and monoclinic nanostructured HfO_2_ thin films were prepared using a nanoarchitectonics approach: sol-gel solutions were spin-coated and then micropatterned by DUV photolithography. Thermal annealing was required to crystallize the HfO_2_, but importantly this did not affect the micropatterning. In parallel, hydrothermally prepared nanoparticles were successfully combined with EPD to directly prepare continuous, dense monoclinic HfO_2_ thin films on ITO-glass substrates with controlled particle size and coating thickness. XRD and TEM data show that the hydrothermal growth time, temperature and pH can be adjusted to control the size and shape of the monoclinic HfO_2_ nanoparticles. The solvent used to prepare colloidal suspensions of the particles and EPD operating parameters were optimized to produce high-quality thin films. GIXRD and SEM data show that this method can be used to deposit nanostructured HfO_2_ films thinner than 200 nm.

## Supplementary Materials

The following supporting information can be downloaded at: https://www.mdpi.com/article/10.3390/nano12142334/s1. Figure S1. (a–c) Digital photographs of coatings prepared by EPD (5 V, 0.7 mA/cm², 90 s) on ITO glass substrates using suspensions of (a) 5 nm, (b) 50 nm, and (c) 100 nm-sized HfO2-NPs dispersed in an equimolar mixture of ethanol and acetylacetone at pH 2; Figure S2. AFM images of the films prepared by EPD; Figure S3. Grazing incidence X-ray diffraction patterns of electrophoretic coatings of (a) 5 nm, (b) 50 nm, and (c) 100 nm sized particles at incidence angles of 0.2, 0.4, 0.6 and 0.8° from the surface. Table S1: Values of roughness for the thin films prepared by EPD.
