# Supplementary material for: Hafnium Oxide Nanostructured Thin Films: Electrophoretic Deposition Process and DUV Photolithography Patterning
Source: Nanomaterials (Basel). 2022 Jul 7;12(14):2334. doi: 10.3390/nano12142334 (PMC9320788; doi:10.3390/nano12142334)
Supplement: Supplementary file 1 [file nanomaterials-12-02334-s001.zip › nanomaterials-1764617-supplementary.pdf]

nanomaterials

MDPI

Article

Hafnium Oxide Nanostructured Thin Films: Electrophoretic Deposition Process and DUV Photolithography Patterning.

Vanessa Proust[1,2,3,*], Quentin Kirscher[4,5], Thi Kim Ngan Nguyen[3,6], Lisa Obringer[4,5], Kento Ishii[2], Ludivine Rault[7], Valérie Demange[7], David Berthebaud[3], Naoki Ohashi[2,3], Tetsuo Uchikoshi[2,3], Dominique Berling[4,5], Olivier Soppera[4,5,*], Fabien Grasset[3,7*]

1 CEA, DES, ISEC, DMRC, Université de Montpellier, F-30200 Marcoule, France
2 Research Center for Functional Materials, National Institute for Materials Science (NIMS), Tsukuba 305-0044, Japan; ishii.kento@nims.go.jp (K.I.); ohashi.naoki@nims.go.jp (N.O.); uchikoshi.tetsuo@nims.go.jp (T.U.)
3 CNRS-Saint Gobain-NIMS, IRL 3629, Laboratory for Innovative Key Materials and Structures (LINK), NIMS, Tsukuba 305-0044, Japan; nguyen.thikimngan@nims.go.jp (T.K.N.N.); david.berthebaud@cnrs.fr (D.B.)
4 Université de Haute-Alsace, CNRS, IS2M UMR 7361, F-68100 Mulhouse, France; quentin.kirscher@uha.fr (Q.K.); lisa.obringer@uha.fr (L.O.); dominique.berling@uha.fr (D.B.)
5 Université de Strasbourg, F-67081 Strasbourg, France
6 International Center for Young Scientists, ICYS-Sengen, Global Networking Division, NIMS, Tsukuba 305-0047, Japan
7 Univ Rennes, CNRS, ISCR UMR 6226, ScanMAT UAR 2025, F-35000 Rennes, France; ludivine.rault@univ-rennes1.fr (L.R.); valerie.demange@univ-rennes1.fr (V.D.)
* Correspondence: vanessa.proust@cea.fr (V.P.); olivier.soppera@uha.fr (O.S.); fabien.grasset@univ-rennes1.fr (F.G.)

	Zone 1<br>5 x 5 µm²<br>(± 0.5 nm)	Zone 2<br>2 x 2 µm²<br>(± 0.5 nm)	Average roughness<br>(± 0.5 nm)
**HfO_2-100**	11.1 nm	6.6 nm	9.0 nm
**HfO_2-50**	16.8 nm	10.3 nm	14.0 nm
**HfO_2-5**	10.6 nm	5.3 nm	9.0 nm

Table S1: Values of roughness for the thin films prepared by EPD.

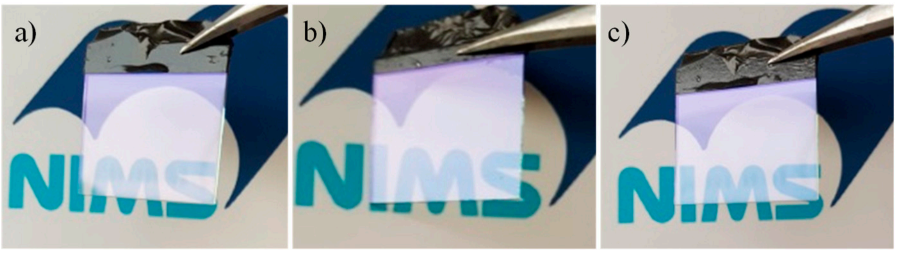

Figure S1. (a–c) Digital photographs of coatings prepared by EPD (5 V, 0.7 mA/cm², 90 s) on ITO glass substrates using suspensions of (a) 5 nm, (b) 50 nm, and (c) 100 nm-sized HfO_2-NPs dispersed in an equimolar mixture of ethanol and acetylacetone at pH 2.

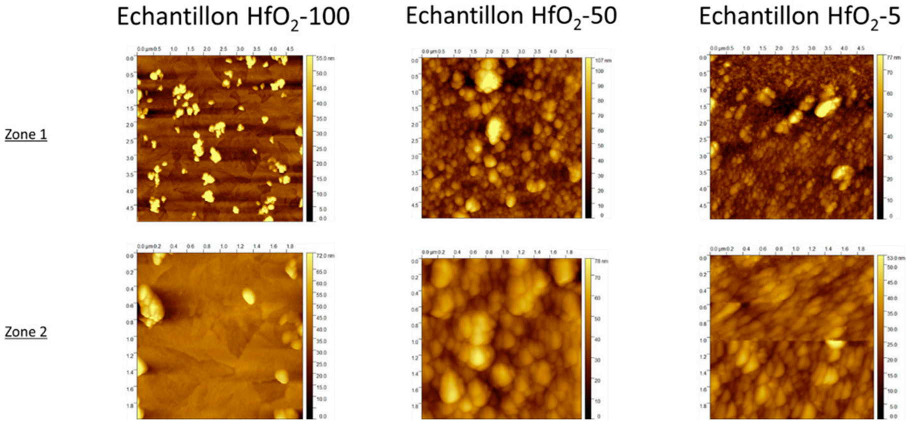

Figure S2. A2M images of the films prepared by EPD.

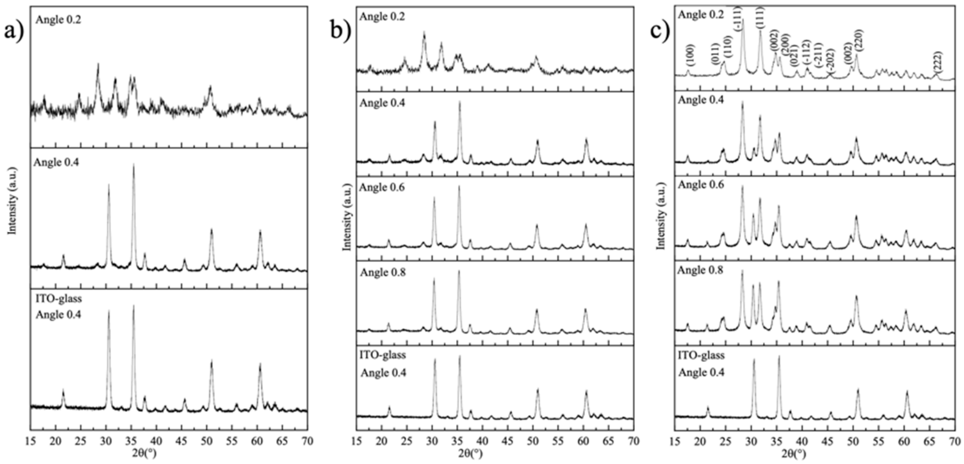

Figure S3. Grazing incidence X-ray diffraction patterns of electrophoretic coatings of (a) 5 nm, (b) 50 nm, and (c)100 nm sized particles at incidence angles of 0.2, 0.4, 0.6 and 0.8° from the surface.
